# Effects of zinc on CarE activities and its gene transcript level in the English grain aphid, *Sitobion avenae*

**DOI:** 10.1093/jis/14.1.67

**Published:** 2014-01-01

**Authors:** Huan-Huan Gao, Hui-Yan Zhao, Jie Yang, Li Zhang, Xiao-Hui Bai, Zu-Qing Hu, Xiang-Shun Hu

**Affiliations:** State Key Laboratory of Crop Stress Biology in Arid Areas, College of Plant Protection, Northwest A&F University, Yangling, Shaanxi 712100, China

**Keywords:** activity, biomarker, expression

## Abstract

As a selective stress, heavy metals play an important role in inducing the adaptive adjustments of insects to changing environments. Carboxylesterase (CarE) is one kind of biomarker that could help us to explore the adaptation mechanism of aphids to heavy metal stress. In this study, CarE activity and gene expression level were investigated in English grain aphids, *Sitobion avenae* (F.) (Hemiptera: Aphididae), exposed to Zn2+ at concentrations of 0, 400, and 1600 mg/kg for 5, 15, 25, and 30 generations. The results showed that the CarE activity was significantly different between different Zn2+ concentrations and different generations. The CarE activity significantly decreased with increasing generations. In the higher generations, the CarE activity was strongly inhibited by the 1600 mg/kg of Zn2+. Realtime quantitative PCR revealed that the CarE gene expression pattern in *S. avenae* was up-regulated under the condition of 400 mg/kg and 1600 mg/kg of Zn2+, but a significant difference was not found after long-term exposure to high concentrations of Zn2+. It was concluded that CarE could be the sensitive biomarker for *S. avenae* response to the presence of Zn2+. In order to adapt to heavy metal Zn2+ stress, *S. avenae* had particular patterns of gene expression under long-term stress.

## Introduction

Because of human activities such as mining, smelting, and other industrial activities, heavy metal pollution has been a problem in nearly every country in the world ( Nriagu 1996 ). In some cases, the pollution has been extensive enough to lead to environmental disasters and ecosystem deterioration ( Sainz et al. 2004 ). In polluted habitats, heavy metals have been found to accumulate in insects through the food chain system ( Väisänen et al. 1993 ; Wang et al. 2005 ). Ecological biomarkers such as developmental period, weight, fecundity, mortality, and insect population number are affected by heavy metals (Ruohomaki et al. 1996; Mousavi et al. 2003 ; Hayford and Ferrington 2005 ).

Environmental stress plays a crucial role during biological evolution and influences the capacity of the insects to respond to environmental changes ( Harrington et al. 2001 ). The English grain aphid, *Sitobion avenae* (F.) (Hemiptera: Aphididae), is one of the most serious pests attacking cereal plants and vec- toring debilitating plant viruses ( Blackman and Eastop 1984 ; Oerke 1994 ). The aphid evolves and adapts to changing environments quickly because of their parthenogenesis and high fecundity. Therefore, *S. avenae* is a good subject for researching adaptation and evolution of insects. Zinc is a common heavy metal in the earth’s crust, and it is present in quantities of about 75 mg/kg soil on average ( Emsley 2001 ). However, at contaminated sites near a Pb/Zn mine, Zn concentrations in the soil reached 800 mg/kg ( Zhuang et al. 2009 ). In the vicinity of the Boleslaw Zn smelter near Olkusz in southern Poland, Zn concentration in the humus layer exceeded 9600 mg/kg ( Stone et al. 2001 ). Therefore, the low and high concentrations of 400 and 1600 mg/kg were used in this study to research the effect Zn on aphids.

As the first warning signals to predict changes in organisms under environmental stress, biomarkers play important roles in ecotoxicological studies ( Vlahović 2012 ). Carboxylesterases (CarE) contribute to neutralize both ester and amide bonds of xenobiotics with low substrate specificity ( Toung et al. 1990 ; Jokanovic 2001 ) and they are sensitive biomarkers to heavy metals ( Migula et al. 1999 ). For example, the CarE activity of female ground beetle, *Pterosthicus oblongopunctatus* , was significantly higher in beetles from five sites along a gradient of heavy metal pollution, but the male beetles did not differ in enzyme activity along the metal gradient ( Stone et al. 2002 ). Zvereva et al. (2003) examined the activity of CarE in the leaf beetle, *Chrysomela lapponica* , from contaminated habitats (Ni and Cu). They indicated that tolerance of nonspecific esterases to heavy metals was higher in leaf beetle populations from contaminated environment than beetles from unpolluted habitats. The CarE activity of *Spodoptera litura* exposed to low-dose Ni (5 mg/kg) for three generations was inhibited, but was induced by high-dose Ni (10 mg/kg) ( Sun et al. 2008 ). In *S. avenae* , the activity of CarE also changed under treatment with Zn for three generations ( Zhang and Zhao 2009 ).

The changes in CarE activity were inevitably accompanied by changes in gene expression level in the receptor organisms under the environmental stress ( Straalen 2003 ; Korsloot et al. 2004 ). It was possible to generate different patterns of gene expression, such that genes were up-regulated, down-regulated, and unal- tered ( Brulle 2010 ), including interactive effects ( Chapman et al. 2006 ). The gene expression of CarE has been measured with qPCR in the field of insect tolerance to pesticides. The CarE gene in cotton aphids ( *Aphis gossypii* ) was overexpressed when associated with organophosphorous insecticide tolerance ( Cao et al. 2008 ). Quantitative realtime PCR showed that the CarE gene was overexpressed in response to beta-cypermethrin in *Musca domestica* ( Zhang et al. 2010 ).

For *S. avenae* , research on CarE has been limited to three generations. However, it is well known that adaptations of species to experimental stress are attributed to the long-term hereditary selection. Therefore, the adaptations of *S. avenae* exposed to heavy metal Zn needs to be studied in more than three generations. Roelofs et al. (2010) proposed that gene expression reaction norms may be important in the evolution of stress tolerance and adaptation to environmental stressors, including heavy metals. However, the genetic expression of CarE in *S. avenae* exposed to Zn has not been examined. As a biomarker, the study of CarE gene expression level is crucial to explore the adaptation mechanisms of *S. avenae* to Zn. Therefore, on the basis of examination of CarE activity, the research of genetic transcript levels will provide more evidence on the hereditary of *S. avenae* exposed to Zn. In this study, *S. avenae* was reared for 30 consecutive generations on wheat, *Triticum aestivum* L. (Poales: Poaceae), treated with Zn. The CarE activity and the relative transcript level of the CarE gene were detected as biomarkers, providing evidence for the evolution of *S. avenae* that are exposed to Zn.

## Materials and Methods

### Aphids and plants treated with zinc

*S. avenae* were collected from the Laboratory of Crop Stress Biology in Arid Areas in the district of Yangling, Shaanxi Province, China, in April 2010.

Dried soil weighing 1 kg was placed in plastic pots (9 × 9 × 10 cm) containing zinc (Zn) as ZnSO4·7H2O at concentrations of 400 mg/kg in three pots and 1600 mg/kg in three pots. Three pots with non-contaminated soil were used as the control. Wheat seeds were then planted in the pots (15 seeds per pot). The plants were reared in a climate-controlled chamber at 20 ± 0.3°C during the day, 18 ± 0.3°C during the night, and 60% RH with a 14:10 L:D photoperiod.

When the wheat grew to the three-leaf stage (code 12 to 13) ( Zadoks et al. 1974 ), 30 first instar nymphs of *S. avenae* were placed on the plants in each pot. When the aphids began to reproduce, 30 first instar nymphs were transferred to new wheat plants treated with Zn in each pot, and continued similarly up to 30 consecutive generations in a climate- controlled chamber. Each pot was maintained in a cage (15 × 15 × 30 cm) made of net and plastic to prevent aphids from moving to other treatments. When nymphs developed into adults, aphids were used to measure the CarE activity and its gene’s transcript level.

### Determination of Zn concentrations in wheat

The concentration of Zn in wheat was detected according to the method of Gao et al. (2012 ). At the two to three-leaf stage, the host plant’s Zn level was determined by homogenizing 1 g of fresh wheat leaf tissue with a mortar and pestle and dissolved in a mixture of HNO3/HClO4 (3:1 v/v) in each treatment for three replicates. Zn was determined by flame atomic absorption spectrophotometry (Hitachi Z-2000, ( www.hitachi.com ).

### CarE activity assay

For each analysis, 30 adult aphids from each pot were collected at the 5th, 15th, 25th, and 30th generations and were used to obtain the crude extract according to Campa-cớrdova et al. (2002). Three replications (three pots) were designed for each treatment. The aphids were homogenized in Na–phosphate buffer (0.1 M, pH 7.0) containing 0.5% Triton X-100 (Prabhakaran and Kamble 1993). The homogenates were centrifuged at 12,000 g for 15 minutes and supernatants were used for biochemical assays.

CarE activity was determined by the method of Van Asperen (1962) , using alpha-naphthyl butyrate substrate. A crude extract of 0.1 mL from each treatment was added to the reaction mixture (4 mM alpha-naphthyl butyrate, 3 mL) and dissolved with phosphate buffer (0.1 mM, PH 7.0). Samples were incubated at 37°C for 30 min and were left for 30 min after adding 1 mL TMB solution (1% fast blue B salt: 5% SDS = 2:5). Then, the assay was carried out under fluorescent light with A600. CarE activity was calculated using alpha- naphthol standard curve and expressed as U. 1 U is the amount of enzyme required to synthesize 1 micromole alpha-naphthol per minute per g protein. The protein concentration was determined according to Bradford (1976) , using bovine serum albumin (fraction V) as the standard.

### Relative transcript level of CarE gene

Gene transcript level was measured by qPCR. To determine PCR efficiency, standard curves were obtained in triplicate for primer of CarE and Actin gene, which seems to be a housekeeping gene, with four-fold dilutions of a standard batch cDNA ( Pfaffl 2001 ). Total RNA was extracted from 30 aphids each from the 5th, 15th, 25th, and 30th generations from each different treatment using the TRIZOL reagent (Invitrogen, Life Technologies, ( www.lifetechnologies.com ) and quantified on a Nanodrop ND-2000 Spectrophotometer (NanoDrop, ( www.nanodrop.com ). Three biological replicates of RNA samples of each treatment group were prepared, and 2 µg of total RNA was reverse transcribed. Second- strand cDNA was amplified and quantified by adding forward and reverse primers specifically for CarE and Actin genes.

The qPCR reactions were performed with a Bio-Rad iCycler (( www.bio-rad.com ) and a SYBR® Green I detection method. The reaction was carried out using Ultra SYBR Mixture kit (CoWin, ( www.cwbiotech.bioon.com ) in iCycler iQ5 Realtime PCR Thermal Cycler (Bio-Rad) in triplicate. The 20 µL reaction system contained 10 µL SYBR Green Master Mix (Applied Biosystems, Life Technologies), 0.5 µmol/L of specific forward and reverse primer, and 2 µL of the diluted cDNA. The following thermal profile was used: 95°C for 10 min and 40 cycles at 95°C for 15 sec followed by 60°C for 30 sec. The gene transcript level was calculated according to the following formula:

Relative transcript level = 2−∆∆CT

$\Delta\Delta = (\text{C}_\text{t;target} - \text{C}_{\text{t;reference}})_{\text{sample}} - \text{C}_{\text{t;target}} - \text{C}_{\text{t;reference}})_{\text{control}}$

### Statistical analysis

Oneway ANOVA (α = 0.05) was used to analyze the Zn concentration in wheat planted in contaminated soil. CarE activity and its gene’s transcript level in *S. avenae* were analyzed with two-way ANOVA (α = 0.05) with generation and concentration as factors. The data were examined for normality and homosce- dasticity of variance using Levene’s test of equality of error variances. The activity in different concentrations (0, 400, 1600 mg/kg) were tested using Student-Newman-Keuls multiple comparisons with SPSS 17.0 statistical analysis package (IBM, ( ww.ibm.com ) for the 5th, 15th, 25th, and 30th generations. Moreover, for treatments with 400 and 1600 mg/kg of Zn, the multiple comparisons test was also conducted with respect to Zn concentration to investigate the effect of Zn on CarE gene relative transcript level in *S. avenae* of each generation.

## Results

### Zn concentrations in wheat

**Figure 1.**
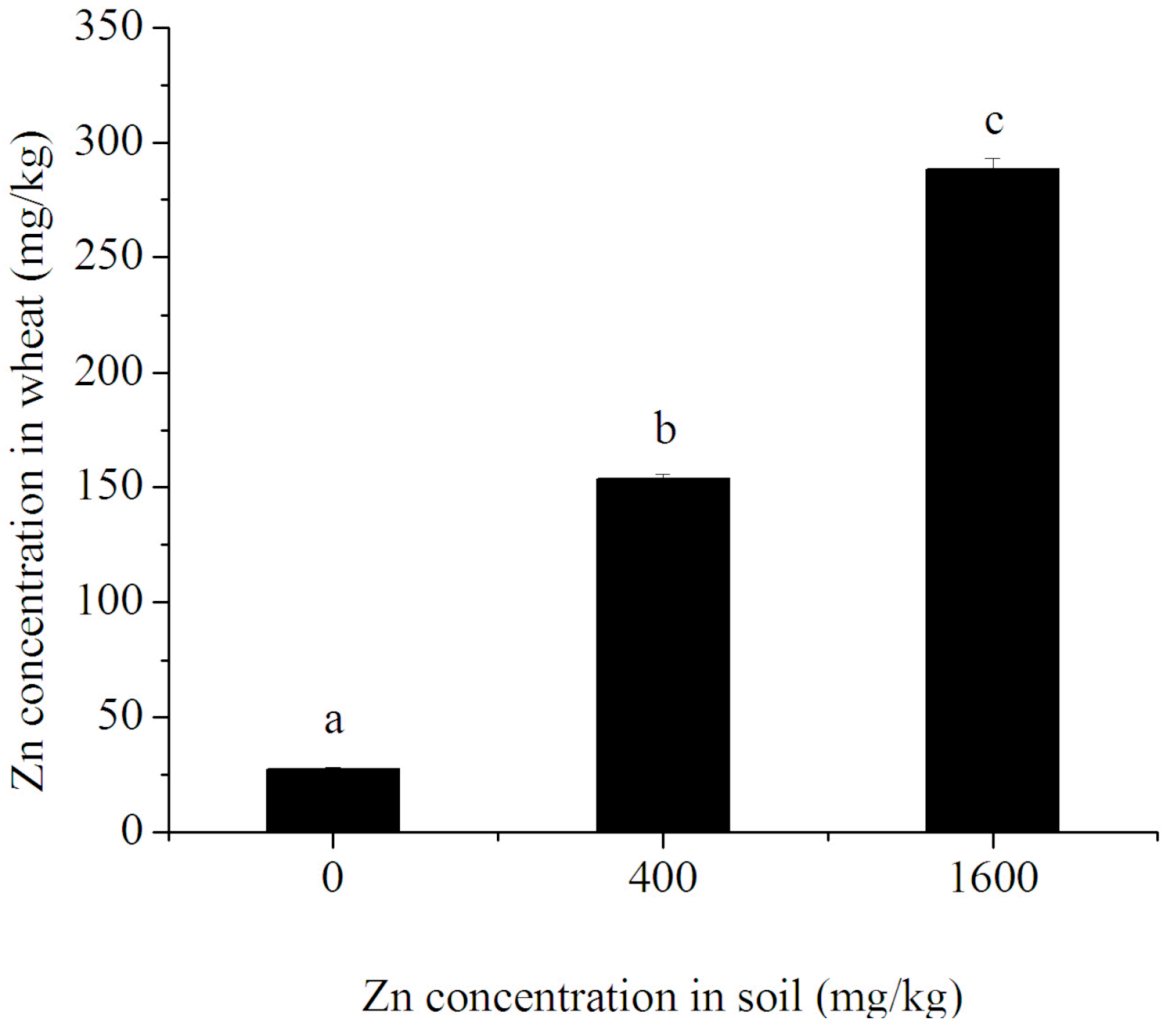
Zn concentration in leaves of wheat planted in soil contaminated with Zn. Different letters (a–c) indicate a significant difference (Student-Newman-Keuls test: *P* < 0.05, following oneway ANOVA). High quality figures are available online.

Zn concentration in leaves of wheat significantly increased with increased Zn concentrations in soil ( *F* = 1922.748, df = 2, *P* < 0.001). As shown in Figure 1 , in the plant in uncontaminated soil, Zn existed at a concentration of 27.664 ± 0.675 mg/kg. When soil Zn concentrations were 400 mg/kg and 1600 mg/kg, Zn was present in wheat leaves in concentrations of 153.981 ± 1.693 mg/kg and 288.496 ± 4.819 mg/kg, respectively.

### CarE activity of *S. avenae*

The result sof two-way ANOVA on CarE activity of *S. avenae* exposed to Zn with two factors (generation and concentration) are presented in Table 1 . It was concluded that the activity was affected significantly by concentration of zinc ( *F* = 141.41, df = 2, *P* < 0.001), generations treated consecutively ( *F* = 14.46, df = 3, *P* < 0.001), and the interaction effect between them ( *F* = 9.61, df = 6, *P* < 0.001). Under each treatment of concentration, the activity significantly decreased with increasing generations treated.

**Table 1.**
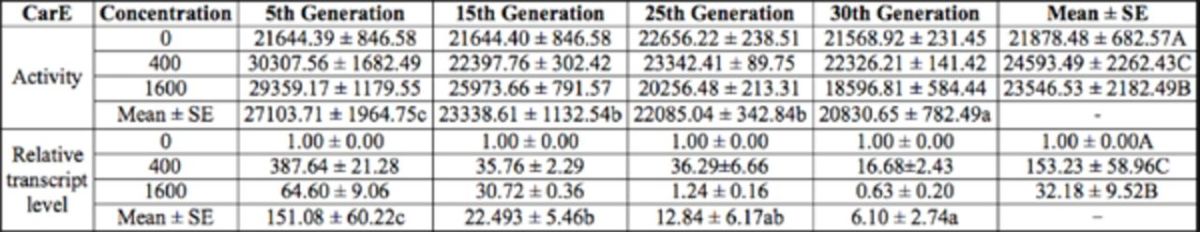
CarE activity and relative transcript level of *Sitobion avenae* under the stress of Zn (mean ± SE).

**Figure 2.**
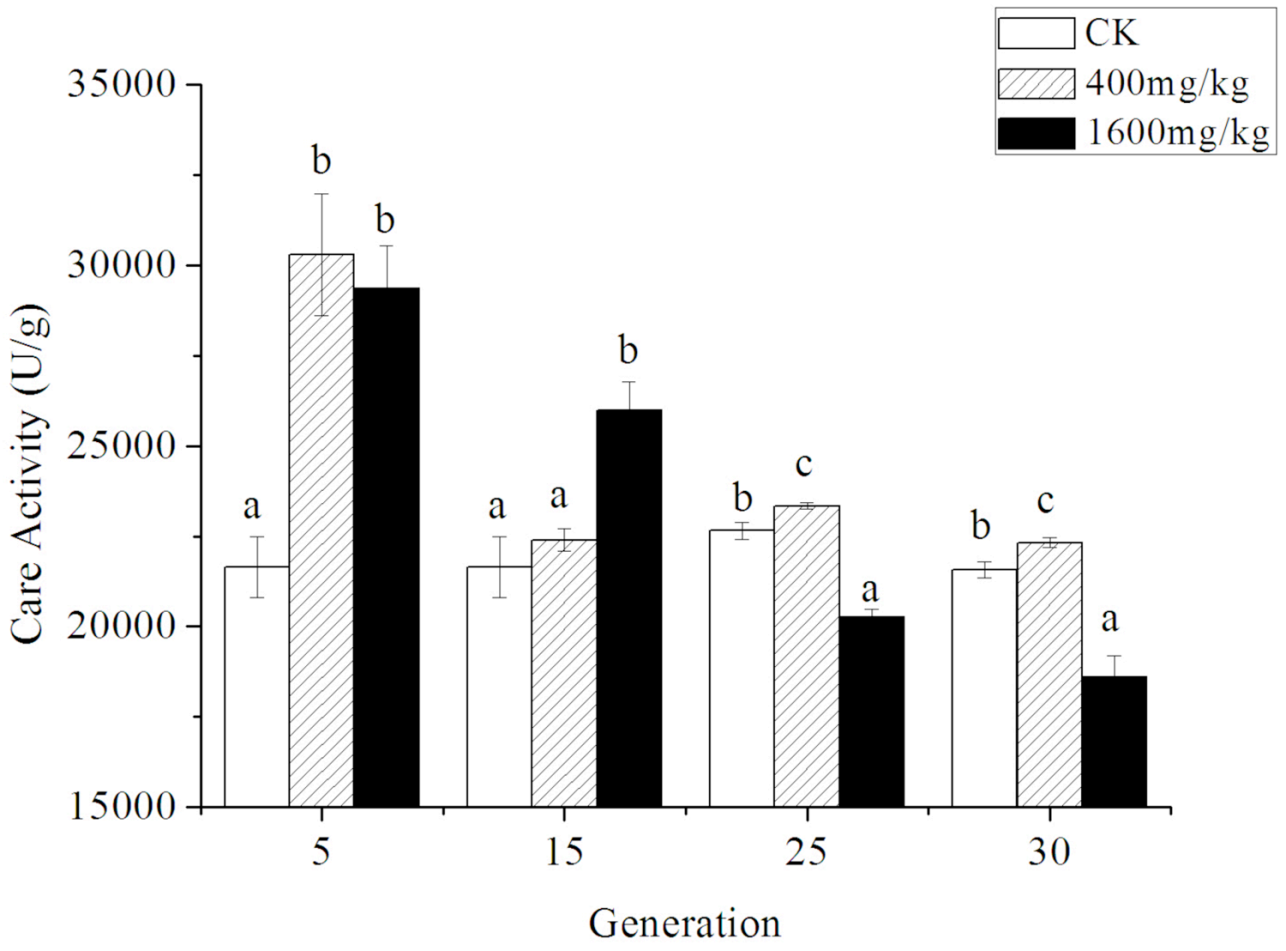
The activity of CarE in adult *Sitobion avenae* exposed to three levels of Zn treatments (CK indicates the control) for 5, 15, 25, and 30 generations. Different letters indicate a significant difference among different concentrations in 5th, 15th, 25th, and 30th generation aphids (Student-Newman-Keuls test: *P* < 0.05, following oneway ANOVA). High quality figures are available online.

The changes in CarE activity in *S. avenae* and the results of oneway ANOVA for different concentrations in each generation are shown in Figure 2 . When the aphids were treated for five generations, the activity of CarE increased to 30307.56 ± 1682.47 U/g and 29359.17 ± 1179.55 U/g in 400 mg/kg and 1600 mg/kg of Zn, respectively, compared to that of control (21644.39 ± 846.58 U/g; *F* = 13.72, df = 2, *P* = 0.006). In the 15th generation, 1600 mg/kg of Zn increased the CarE activity significantly (25973.66 ± 791.57 U/g; *F* = 11.19, df = 2, *P* = 0.009). However, there was not a significant difference between populations under the stress of 400 mg/kg of Zn and the control. Though 400 mg/kg of Zn could increase the CarE activity (except for the 25th generation), CarE activity was affected significantly negatively by Zn at the concentration of 1600 mg/kg in the 25th generation (1600 mg/kg, 20256.48 ± 213.31 U/g; control, 22656.22 ± 238.51 U/g; *F* = 71.32, df = 2, *P* < 0.001) and 30th generations (1600 mg/kg, 18596.81 ± 584.44; control, 21568.92 ± 231.45; *F* = 582.92, df = 2, *P* < 0.001). So, it was concluded that, the effect of Zn would decrease with the increasing of generations treated, and the CarE activity was inhibited evenly by the 1600 mg/kg of Zn at high generations.

### Relative transcript level of CarE gene

The relative transcript level of the CarE gene in *S. avenae* exposed to Zn and the results of two-way ANOVA with two factors (genera The row of ‘Mean ± SE’ of CarE activity and relative transcript level shows the mean of all values in the column. Different lowercase letters (a–c) in the line indicate the significance in different generations of *Sitobion avenae* . The “Mean ± SE” column shows the mean of all values in the row. Different capital letters (A–C) indicate a significant difference in different concentrations of Zn (Student-Newman-Keuls test: *P* < 0.05, following two-way ANOVA) tion and concentration) are shown in Table 1 . The role of CarE regulation in Zn tolerance was investigated by means of qPCR. The mean normalized relative expression values were calculated between exposed and non- exposed *S. avenae* among the three replicates. They were affected significantly by concentration of Zn ( *F* = 317.894, df = 2, *P* < 0.001), number of generations treated consecutively ( *F* = 235.998, df = 3, *P* < 0.001), and the interaction effect between them ( *F* = 217.721, df = 6, *P* < 0.001). Similar to the changes in activity, the expression level decreased significantly with increasing concentrations of Zn and increasing number of generations treated by Zn.

**Figure 3.**
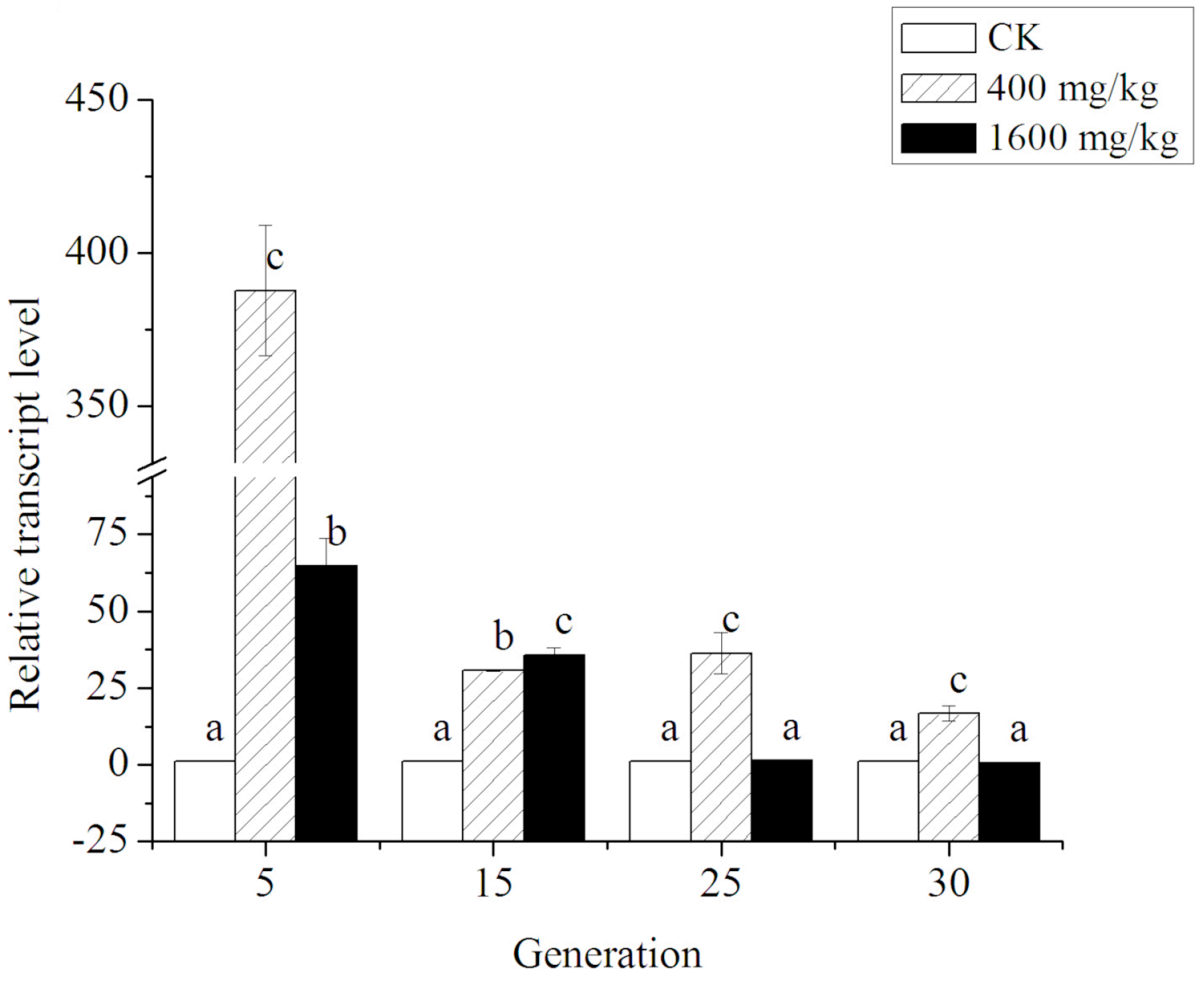
The relative transcript level of CarE in adult *Sitobion avenae* exposed to three levels of Zn treatment (CK indicates the control) for 5, 15, 25, and 30 generations. Different letters indicate a significant difference among different concentrations in 5th, 15th, 25th, and 30th generation aphids (Student-Newman-Keuls test: *P* < 0.05, following oneway ANOVA). High quality figures are available online.

CarE gene expression patterns in *S. avenae* upon exposure to Zn and the results of oneway ANOVA among different concentrations in each generation are shown in Figure 3 . When the aphids were exposed for 5 generations, the expression of CarE was up-regulated by 387.64 ± 21.28 and 64.60 ± 9.06 fold under 400 and 1600 mg/kg of Zn, respectively, compared to the control (1.00 ± 0.00) ( *F* = 240.99, df = 2, *P* < 0.001). Then, after 15 generations, 1600 mg/kg of Zn induced the transcript of CarE gene more significantly than 400 mg/kg ( *F* = 197.12, df = 2, *P* < 0.001). In the 25th and 30th generations, 400 mg/kg of Zn increased the CarE gene transcript level significantly (25th generation: *F* =27.85, df = 2, *P* = 0.001; 30th generation: *F* = 42.48, *df* = 2, *P* < 0.001), but no significant difference between *S. avenae* from the control and the population polluted by 1600 mg/kg of Zn was found. Although the expression of CarE was not affected significantly by 1600 mg/kg of Zn after 30 generations, it was expressed at a slightly lower level (0.63 ± 0.20 fold) compared to the control, like the result of activity.

Therefore, it is concluded that under the conditions of 400 mg/kg and 1600 mg/kg of Zn, the CarE gene in *S. avenae* had the same pattern of gene expression. It was up-regulated compared to the control population, but not significantly in high concentrations, and decreased with the increasing of exposure time. The changing role of gene expression was in accordance with that of CarE activity in *S. avenae* exposed to Zn.

## Discussion

The changes in development and reproduction of *S. avenae* exposed to Zn was researched by Zhang and Zhao (2009) . The organism adapt- ing to a contaminated environment is necessary for surviving in ever-changing environments. However, the ability of adaptation depends mainly on effective mechanisms of detoxification ( Jokanovic 2001 ). The role of esterases in neutralizing xenobiotics has been found by many researchers ( Blackstock 1984 ; Bogaerts et al. 2009 ).

In this work, CarE was selected to be the biomarker to explore the adaptation of *S. avenae* to exposure to Zn. We compared the general activity and gene expression pattern of CarE in populations of *S. avenae* from environments contaminated by Zn. When the aphids were exposed to Zn for 5 generations, the activity of CarE increased compared to that of the control and decreased with increasing generations. In the high generations (25 and 30 generations), the CarE activity was inhibited by 1600 mg/kg of Zn. In other research, similar results were found. Vlahović et al. (2012) concluded that esterases in *Lymantria dispar* showed great sensitivity to low cadmium concentrations during acute and chronic treatments. Their activities during short-term exposure and after recovery significantly depended on cadmium concentrations. Larvae of beetles ( *Poecilus cupreus* ) exposed to Zn at high concentrations had lower CarE activity compared to the control ( Wilczek 2003 ). They confirmed that in studying enzyme activity under metal stress one should consider the life-stage of insects and the type of heavy metal.

Moreover, in this study, qPCR of *S. avenae* exposed to Zn revealed that, under the stresses of 400 mg/kg and 1600 mg/kg of Zn, the CarE gene of *S. avenae* had the same pattern of gene expression, which was up-regulated compared to the control population. However, the significant difference of relative transcript level of the CarE gene was not found after long-term exposure to a high concentration of Zn. The variation in general activity and gene expression of CarE decreased with increasing concentration and exposure time. This phenomenon could be explained by the adaptation mechanism under the long-time exposure to Zn. Insects could adapt to the toxicity of low- level pollutions through equilibrium mechanisms and metabolism. However, physiological confusion would occur under high-level stress ( Kay 1985 ; Dallinger et al. 1987 ). In our experiment, 5th and 15th generation aphids responded to Zn toxicity by increasing CarE activity and upregulation of the CarE gene. However, under the long-term exposure to Zn (30 generations), a new advanced pattern of adaptation was probably induced by a high-concentration of Zn due to confusion in the physiology of *S. avenae* .

In research on the transcriptional activity of a number of various genes modulated by heavy metals, analysis for expression levels of Metallothionein (MT) genes has been widely performed, with isoforms of MT genes displaying time- and dose-dependent upregulation of expression in various phyla. They are strongly induced by heavy metals, such as Hg, Cu, Cd, and Zn ( Brulle et al. 2010 ). In addition, microarray-based transcriptomics has the potential to be a tool of choice in ecotoxicology ( Roh et al. 2009 ) and is anticipated to play an important role within a tiered framework of environmental diagnostics ( Ankley et al. 2006 ). The adaptive mechanism of *S. avenae* exposed to heavy metals needs to be explored through transcriptomics in the future.
